# Accelerating the translation of findings from the MoTrPAC study to benefit clinical care: a qualitative analysis

**DOI:** 10.1186/s12875-025-03030-6

**Published:** 2025-10-27

**Authors:** Katherine A. Collins-Bennett, Kandice R. Lacci-Reilly, Aylin Aguilar, Jerome L. Fleg, Stephanie M. George, Joseph A. Houmard, Kim M. Huffman, Neil M. Johannsen, Daniel H. Katz, Julia Narendra, Shlomit Radom-Aizik, Lauren M. Sparks, Ashley Xia, Dan M. Cooper

**Affiliations:** 1 Duke Molecular Physiology Institute, Duke University School of Medicine, Durham, NC 27708 USA; 2 Department of Population Health Sciences, Duke University School of Medicine, Durham, NC 27708 USA; 3 Department of Social Sciences & Health Policy, Wake Forest School of Medicine, Winston-Salem, NC USA; 4 National Heart, Lung, and Blood Institute, National Institutes of Health, Bethesda, MD USA; 5 Office of Dietary Supplements, National Institutes of Health, Bethesda, MD USA; 6 Department of Kinesiology, Human Performance Laboratory, East Carolina University, Greenville, NC USA; 7 Division of Rheumatology and Immunology, Department of Medicine, Duke University School of Medicine, Durham, NC USA; 8 Preventative Medicine, Pennington Biomedical Research Center, Baton Rouge, LA USA; 9 Department of Medicine, Stanford University School of Medicine, Stanford, CA USA; 10 UC Irvine Pediatric Exercise and Genomics Research Center, University of California - Irvine, Irvine, CA USA; 11 Translational Research Institute AdventHealth, Orlando, FL USA; 12 National Institute of Diabetes and Digestive and Kidney Diseases, National Institutes of Health, Bethesda, MD USA; 13 Institute for Clinical and Translational Science, University of California - Irvine, Irvine, CA USA

**Keywords:** Dissemination, Implementation, Exercise intervention, Physical activity, Reflective thematic analysis

## Abstract

**Background:**

The Molecular Transducers of Physical Activity Consortium (MoTrPAC) is a large-scale research study aimed at elucidating the effects of exercise training on the molecular mechanisms underlying the health benefits of exercise and physical activity. To take a first step toward achieving a goal of rapid dissemination, a qualitative analysis among frontline clinicians was conducted to identify the perceived clinical relevance of exercise research, MoTrPAC discoveries, and optimal ways to disseminate these results to key stakeholders.

**Methods:**

A convenience sample of 12 clinicians in internal medicine, family medicine, and emergency medicine agreed to participate in one-on-one interviews. Interviews were conducted over the phone by a member of the Wake Forest Qualitative and Patient-Reported Outcomes (Q-PRO) Shared Resource. Transcripts were stored and coded in ATLAS.ti version 24 software. Two Q-PRO members developed code summaries, which were synthesized into themes and organized using principles of reflexive thematic analysis.

**Results:**

Clinicians (*n* = 12) were predominantly male (*n* = 7), non-Hispanic White (*n* = 5), located in California (*n* = 10), and practiced in a Primary Care/Family Medicine setting (*n* = 6). Clinicians reported underuse of exercise testing due to provider-level and patient-level barriers. While they valued exercise research, they emphasized the need for clear, practical takeaways and preferred direct dissemination strategies.

**Conclusions:**

These interviews highlighted the variable nature of exercise research dissemination and implementation and are the first steps toward shaping the dissemination of valuable scientific discoveries from the MoTrPAC study.

**Supplementary Information:**

The online version contains supplementary material available at 10.1186/s12875-025-03030-6.

## Background

Extensive scientific evidence supports regularly engaging in physical activity (PA) and exercise to improve overall health and reduce the risk of chronic diseases [1]. Although the health benefits of being physically active are well recognized, the cellular and molecular mechanisms that link PA to health have not been fully elucidated. The limited mechanistic understanding of the health benefits of PA throughout the lifespan restricts clinical applications of PA across various demographic and clinical factors, as well as disease states [2]. Funded by the National Institutes of Health (NIH) Common Fund, the Molecular Transducers of Physical Activity Consortium (MoTrPAC) is designed to develop a compendium of molecular transducers that link exercise to health (https://motrpac.org/) [3]. By determining the acute and chronic physiologic and molecular effects of exercise (endurance or resistance) training in healthy children and adults across age, sex, race, and ethnicity, MoTrPAC intends to fill this research gap. In addition to human studies, MoTrPAC includes preclinical studies that have begun to identify the acute and chronic physiologic and molecular effects of endurance exercise training across multiple organ systems [4–11]. 

However, despite the potential for important translational findings from MoTrPAC, many clinicians struggle to integrate exercise testing and recommendations into routine clinical care. Thus, there is a need for research studies, such as MoTrPAC, to identify and implement strategies to accelerate the integration of research discovery into clinical applications to benefit health. Still, ambitious scientific initiatives risk limited clinical impact when real-world implementation barriers are overlooked. This has led to a large gap between research discovery and the application of evidence into clinical practice, often as prolonged as 17 years [12]. This discovery-to-practice gap became more salient during the COVID-19 pandemic when rapid translation and implementation of biomedical discovery were critical. The discovery-to-practice gap is most apparent among populations from disadvantaged communities [13] and dampens the public health benefit of scientific discoveries [14], such as those that may arise from the MoTrPAC study.

To address and shorten this lag in the clinical application of research findings for the MoTrPAC study, a working group was formed within the MoTrPAC research network to identify strategies to disseminate and rapidly apply MoTrPAC discoveries to clinical settings. As a first step toward achieving a goal of rapid dissemination, the working group conducted an exploratory qualitative analysis among a convenience sample of frontline clinicians to (1) explore clinician experiences with and perceptions of exercise testing; (2) assess the research translation process as a whole; and (3) identify the perceived clinical relevance of MoTrPAC discoveries and optimal ways to communicate these results to key stakeholders. Findings from this conceptual study will (1) inform future sampling in larger, more diverse samples implementing both qualitative and quantitative analyses to determine optimal ways to rapidly disseminate MoTrPAC study findings; and (2) guide the development of communication strategies for clinicians and the public to learn about key discoveries from MoTrPAC, and possibly other similar studies.

## Methods

### Study design and sample

An exploratory qualitative study design was used to uncover experiences with exercise testing and perceptions of the research translation process and MoTrPAC findings [15]. This study included a convenience sample of frontline, full-time clinicians. Purposive sampling was selected because it allowed for cost-effective and timely exploration of our research question. As such, we reached out to physicians in our region who were known to us as primary care clinicians whose practice involved general and/or emergency care, and their patients consisted of a general and wide range of diseases and conditions. We had no a priori information regarding any of the individual clinician’s knowledge or experience in exercise or sports medicine. We contacted a total of 19 clinicians in internal medicine, family medicine, and emergency medicine via email. Twelve of the 19 agreed to participate in the one-on-one interviews. Interviews were reviewed after completion to evaluate thematic saturation and consider any necessary changes to the interview protocol. Additional recruitment efforts ceased when thematic saturation was determined after twelve interviews. Thematic saturation is used in qualitative research as a criterion for discontinuing data collection and/or analysis [16], meaning additional interviews would provide no new information and will be redundant to data already collected [17]. The study was determined to be Exempt by the University of California (UC) Irvine and Wake Forest University (IRB00100858) Institutional Review Boards given the interviews offered no risk to participants and did not include private identifiable information, thus informed consent was not required. Each clinician participant received $50 for participation.

### Definitions

*Exercise*, in general, is a subset of physical activity that is planned, structured, and repetitive bodily movement done to improve or maintain one or more components of physical fitness [18]. *Exercise training* is repeatedly exercising with a specific purpose or end goal in mind across various periods of time, generally used to improve or maintain health, cardiovascular endurance, and/or strength [18]. *Exercise testing* is a noninvasive procedure that provides diagnostic and prognostic information and evaluates an individual’s capacity for dynamic exercise [19]. 

### Data collection

The MoTrPAC Clinical Applications Working Group (CAWG) consulted with the Qualitative and Patient-Reported Outcomes (Q-PRO) Shared Resources of the Atrium Health Wake Forest Baptist Comprehensive Cancer Center (https://school.wakehealth.edu/research/Q-PROSharedResources) to develop a semi-structured interview guide to determine how clinicians might apply MoTrPAC findings into their practices. The Wake Forest Center has an extensive track record in robust semi-qualitative and qualitative research, particularly in collaboration with large multi-center trials [20–22]. The semi-structured interview guideline is shown in Table 1. The interview was divided into three major areas, (1) Clinical Practice, (2) Research Engagement, and (3) Research Translation. The guide was revised once to gather more context on research translation and information seeking.

**Table 1:** Interview topics from the semi-structured interview guide

Topic	Content	Example Question
Clinical Practice	• Experience with exercise testing (ET)	*How often do you refer patients for ET?* *What specific patient populations or demographics can benefit the most from ET in your care setting?*
	• Perceptions of ET	
	• Barriers to incorporating ET	
Research Engagement	• Experiences with and perceptions of exercise research	*Tell me about your experiences reviewing exercise research.* *As a clinician*,* how important is it to you to familiarize yourself with current exercise research?*
	• Value of research familiarization	
	• Pathways for learning about emerging research	
	• Preferences for learning about MoTrPAC findings	
Research Translation Process	• Translation facilitators	*What can researchers do to make exercise research more easily incorporated into clinical practice?*
	• Translation barriers	
	• Perceived applicability of MoTrPAC findings to clinical practice	

Participants were recruited by email from members of the study team using a standardized invitation to participate. Each participant was emailed a concise overview of the MoTrPAC study that was briefly reviewed at the beginning of the semi-structured interview.

### Study setting

The study was conducted across multiple clinical sites, including hospital systems in three states. Interviews took place from October 2023 through February 2024. Interviews were conducted over the phone by author KRL, a member of the Q-PRO team, who holds a PhD in public health and has extensive qualitative research experience. The interviewer had no established relations with the participants prior to the interviews. Interviews lasted on average 23 min (range: 18 to 34 min) and were audio-recorded and transcribed verbatim using a professional transcription service.

### Data analysis and management

Transcripts were reviewed and compared to the audio files by Q-PRO to ensure accurate transcription. Two Q-PRO researchers reviewed the transcripts and developed a codebook to capture concepts found in the data. Both inductive (using ideas that emerged from the interviews) and deductive (using concepts from the research question and interview guide) coding strategies were used to develop the codebook.

Transcripts were stored and coded in ATLAS.ti version 24 software [23]. To enhance rigor, two Q-PRO researchers independently coded the textual data and met weekly to compare codes. Discrepancies in coding were discussed and resolved iteratively, reflected in codebook updates based on discussions of code meanings and applications. Text segments were reviewed by code and summarized. Code summaries were synthesized into themes and organized using principles of reflexive thematic analysis [16, 24]. 

## Results

Clinicians (*n* = 12) were predominantly male (58%), non-Hispanic White (42%), located in California (83%), and practiced in a Primary Care/Family Medicine setting (50%) (Table 2). We found four overall themes from the semi-structured interviews: (1) exercise testing is an underutilized resource with many provider-level barriers to utilization; (2) patients face access and acceptability barriers to undergoing exercise testing; (3) despite low engagement with exercise research, many clinicians value exercise research and translating findings into clinical practice; and (4) clinicians value the potential applicability of MoTrPAC findings to their clinical practice, while emphasizing the need for clear and concise recommendations communicated directly to clinicians.

**Table 2:** Sample demographics

	*N*	Percent
Practice Setting		
*Primary Care/Family Medicine*	6	50%
*Emergency Medicine*	4	33%
*Internal Medicine (Hospitalist)*	1	8%
*Pediatrics*	1	8%
Location		
*California*	10	83%
*North Carolina*	1	8%
*Florida*	1	8%
Gender		
*Male*	7	58%
*Female*	5	42%
Race/Ethnicity		
*Non-Hispanic White*	5	42%
*Hispanic/Latino*	4	33%
*Asian*	3	25%

### Theme 1: exercise testing is an underutilized resource with many provider-level barriers

Many clinicians in the study did not have experience using exercise testing with patients outside of evaluations for immediate cardiac risk, despite noting the value of the objective measure and potential benefit to patient care.

*“Generally*,* because my practice [is] mostly in the hospital*,* we’re not referring patients that are hospitalized for exercise testing straight up that frequently*,* but at the time of discharge*,* we might refer them for exercise testing for further follow up by their cardiologist or primary care doctor.” (P04*,* Male Hospitalist)*.

Nearly all clinicians shared that they only refer patients to exercise testing if they present with elevated cardiovascular risk. Clinicians in primary care and emergency medicine settings indicated that they used exercise testing primarily to rule out cardiac causes of chest pain or address specific health conditions, such as respiratory issues or atherosclerotic cardiovascular disease, typically targeting middle-aged individuals with relevant risk factors. Several clinicians indicated specific patient populations that benefit from stress testing, including those with elevated cardiovascular risk (e.g., angina, sleep apnea, obesity, or smokers). They also specified that exercise testing in the emergency department is particularly useful for those with limited access to a cardiologist.

*“I think the patients that benefit the most from exercise testing*,* stress testing*,* [in the] emergency department*,* are the ones with difficulties with access to care. Patients that don’t have insurance. Or patients that*,* for whatever reason don’t have easy access to their primary care. Lower medical literacy – those kinds of patient populations that sort of on their own*,* may not actually ultimately get that test if we don’t provide it for them real-time*,* while they’re in the emergency department.” (P12*,* Female ED Clinician)*.

A few clinicians shared reasons why exercise testing can be valuable in clinical practice, such as objectivity, added motivation, and added confidence in exercise abilities (i.e., self-efficacy). They felt exercise testing and training could provide insight into the progression of disease and the potential impact of activity or exercise on changing the disease course. Moreover, they felt exercise testing and training could significantly influence patient care, offering reassurance or guiding further management, particularly in cases where chest pain may be non-cardiac in origin. Some clinicians highlighted the limitations of exercise testing including, (1) it is not a definitive test for ruling out a cardiac event; and (2) the use of exercise testing as an objective tool is likely less common for low-income communities who may not prioritize this type of testing for routine or preventative purposes.

Clinicians described various barriers to using exercise testing in their practice settings, including logistics, feasibility, and provider knowledge. Many discussed logistical concerns with incorporating exercise testing into primary and emergency care settings, such as access to testing labs and the ability to refer patients for exercise testing.

*“I don’t have any options for referring people out for exercise testing*,* in terms of endurance or strength*,* where I would get back feedback*,* nor do I have any guidelines about what to do with that information if I got it back.” (P03*,* Male PCP)*.

Similarly, a handful of clinicians described limited availability of resources and staff to perform testing. One clinician indicated logistical concerns related to patients’ medication histories and the accuracy of exercise tests. Several clinicians shared that competing demands and limited time in both primary care and emergency medicine settings may impact their ability to incorporate additional testing or information during visits. One clinician specified that in emergency settings, patients are not always admitted or seen in the observation area due to capacity challenges, which can delay exercise testing.

Last, a handful of clinicians indicated that some practitioners are not trained or do not have the knowledge of how to use exercise testing for non-cardiac purposes.

*“Like how do we teach these people that they also need to do cardiopulmonary testing? At least for my specialty*,* that is not something that I would prescribe*,* because we haven’t been taught or we [don’t] have any studies that [show] it would be beneficial to do that.” (P07*,* Female ED Clinician)*.

### Theme 2: patients face access and acceptability barriers to undergoing exercise testing

Clinicians described various patient-level barriers that may impact acceptability, interest, or access to exercise testing, including the ability or motivation to complete a treadmill-based test.

*“The patient acceptance of the test*,* some people simply want to lay on a table*,* and have an IV*,* and get a pharmacologic stress and not have to exercise*,* so there’s some people who either don’t have the motivation to even walk on a treadmill for six to eight minutes. Then finally there are some people for other physiologic problems – they’ve got bad arthritis*,* they’ve had a prior stroke*,* they’re not candidates – where they might be a candidate to exercise*,* they’re not a good candidate for a walking stress test*,* so it’s not a one size fits all study is the thing.” (P10*,* Male PCP)*.

A few clinicians indicated that patient motivation can be a barrier when seeking additional tests/care or following additional guidance for more specific exercise recommendations. One clinician explained that doing routine activities like blood work can be a struggle for numerous reasons, including costs and health literacy. Another clinician contemplated environmental factors that patients may face as a barrier to exercise, noting that practitioners should also consider inequitable access to safe places to exercise. Finally, one clinician highlighted culturally specific considerations for their patients who are immigrants, non-English speaking, have transportation barriers and are largely low-income, noting that exercise testing should be “culturally acceptable and economically feasible.” *(P06*,* Female PCP)*.

Several clinicians also considered the economic restrictions for reimbursement around exercise testing and the implications for uninsured and underinsured patients.

*I’m not sure about [costs]*,* but I imagine that if the person doesn’t have the appropriate insurance that it would be difficult to obtain a test like that under no cost. I could see it being expensive.” (P01*,* Male ED Clinician)*.

A few emergency department clinicians explained that insurance status may dictate access to exercise testing and their decision-making around admissions. Another clinician indicated that pharmacological exercise tests, in particular, are costlier.

### Theme 3: despite low engagement with exercise research, many clinicians value exercise research and translating findings into clinical practice

Exposure to exercise research was generally limited, often due to the scope of clinicians’ typical journals and/or lack of specific training, with some attributing this to their field of practice or role. Despite varying levels of engagement, many clinicians acknowledged the importance of familiarizing themselves with exercise research for advancing patient care. However, some rate the importance of exercise testing lower due to existing challenges in encouraging patients to exercise or the perceived applicability to their practice.

Nearly all clinicians had limited experience reviewing exercise research. Some attributed their low engagement to the scope of their typical journals or the accessibility and timeliness of exercise literature. A handful of clinicians also attributed their limited experience to their field of practice or role and that exercise research was limited, in that the literature predominantly involved a narrow patient population.

*“I would say that most of my experience with exercise testing research would be reports of studies to show the benefits of exercise in the population and the fact that basically most Americans don’t get enough exercise.” (P08*,* Female PCP)*.

Clinicians were asked how important familiarizing themselves with the current exercise research was to them. Most clinicians felt that exercise research could be “very important” to advancing patient care, despite the aforementioned barriers. A few explained that since exercise is a determinant of health, understanding exercise in more detailed ways is important.

*“Well*,* I feel like it’s very important because we know that exercise is one of the key determinants of one’s health status. Diet and exercise*,* sleep*,* I mean*,* these basic lifestyle issues*,* we know are much more important for one’s health than usually taking a medication. I feel very strongly that as part of our job*,* especially in primary care and family medicine*,* is preventative healthcare*,* and prevention means review of lifestyle issues*,* and being up to date on the latest recommendations of what works. What’s the evidence of what will make a difference to improve or prevent various health conditions.” (P06*,* Female PCP)*.

A handful of clinicians spoke about the value of applying “up-to-date” research to patients and colleagues. One clinician described the value of exercise research for specific patient populations (i.e., patients with cardiovascular risk). Others, however, rated exercise research lower, considering the challenges they already face with getting patients to exercise.

*“I think that*,* again*,* my perspective is coming from the very basics of even if I could get my patients to – my patients with diabetes or hypertension – to engage in the recommended amount of cardiovascular activity*,* 150 minutes a week*,* then that’s*,* I think*,* really a basic goal. Anything beyond that*,* I think*,* is extra or additional.” (P02*,* Male PCP)*.

Clinicians described the translation process as variable, with some institutions updating policies frequently based on emerging evidence, while others do so less often, typically every few years. Factors influencing translation included the credibility of research, specificity of recommendations, and feasibility of implementation. Barriers included communication challenges, patient access, and logistical constraints hindering the translation process. Facilitators included support from reputable organizations, dissemination efforts targeting both clinicians and patients, and the availability of technical support and funding.

Clinicians in the study also considered that study factors like the specific, impact, and equity considerations of the findings could enhance research translation, as well as the feasibility of dissemination and incorporating the findings into practice (i.e., support for dissemination through partnerships, patient-facing dissemination). See Table 3 for a summary of these insights.

**Table 3:** Research translation barriers and facilitators

Facilitators	Barriers
*Study Design*: *“I think if you have some sort of credibility*,* you’re coming from a lab that has published good papers and people see that*,* and they see that you’re publishing a certain paper. The paper has good methodology*,* and it’s peer-reviewed and all of these things*,* I could imagine that that’s how certain clinicians would start using it in practice. […] I think that the overall – if the paper is robust research*,* that it’s scientifically sound*,* and it has a good sample size*,* if the research itself is directly applicable to our work*,* that would – that obviously makes sense to translate it into clinical practice*,* and if you trust the source*,* so I think all of those things play together.”*	*Patient Access*: “*Exercise just for overall wellness and health may not be reimbursable. If there’s some identified deficit*,* then it’s probably going to be covered under whatever sign*,* symptom*,* or deficit is there. We don’t get a lot of preventative coverage from insurance for prevention of certain conditions.”*
*Patient-Facing Information*: *“Certainly*,* advertising to patients is effective marketing. Right? Like so when patients hear things*,* they’re gonna bring ‘em back to the clinician*,* and so*,* obviously*,* there’s some responsibility there in making sure that they’re hearing things that are helpful and true. I think*,* yeah*,* even marketing to patients*,* I think*,* helps to disseminate information.”*	*Communication*: *“Sometimes*,* they are using verbiage that’s unique to that field. As a rule*,* I feel that it’s pretty understandable*,* but there are times that*,* like I said*,* there’s verbiage that’s unique to that field and that not something that we typically use in primary care.”*
*Feasibility*: *“Well*,* I think if there were specific recommendations to give to patients. Then*,* an easy way to maybe have it be part of their discharge instructions. Or something that maybe nursing went over them when they were discharged—that would be extremely helpful. It would just have to be in a way that was really easy to use and not time-consuming*,* that would pull us away from the other tasks that we had to do.”*	*Feasibility*: *“Time and money. It’s basically time and money. Because primary care clinicians are very*,* they’re very overextended*,* they’re very burnt out. They’re trying to really accomplish so many things with their patients in a given day. Most primary care clinicians are seeing 20 or 25 patients a day. Each one of these patients has three to five problems that they’re bringing up. […] Be very mindful of adding another task on top of their very long to do list on a daily basis.”*
*Dissemination Support*: *“At the end of the day*,* a lot has to do with sharing information. Some way to get it out there*,* either if it’s a video*,* social media*,* using some sort of widely known society. or example*,* for emergency physicians*,* most of them attend ACEP*,* which is American College of Emergency Physicians*,* or SAEM*,* the Society of Academic Emergency Medicine are the big ones. If you had any sort of presence there*,* or any of these researchers are lecturing and making this available for emergency physicians to think about*,* I think that also would put it more in the front burner for physicians to think about*,* emergency physicians to think about.”*	
*Equity Considerations*: *“The other part of it*,* though*,* is how that translates to our specific patient population. […] We have certain characteristics of our patients who the majority are primarily Spanish speaking. The majority have Medicaid coverage or are underinsured and*,* therefore*,* access to resources is dependent on that coverage. Are these results applicable in real life to our patients? Are they able to access these resources? That tends to be how we*,* here*,* think about new data*,* new recommendations*,* new evidence. One*,* would it make sense to our patients just at large*,* and two*,* does it make sense to our specific patients that we see here?”*	
*Specificity and Impact*: *“I think clearly written results and recommendations is probably the best thing. We need to be able to put results in terms that lay people who are not in medicine will understand.”*	

### Theme 4: clinicians value the potential applicability of MoTrPAC findings to their clinical Practice, while emphasizing the need for clear and concise recommendations communicated directly to clinicians

Clinicians expressed curiosity and optimism about the MoTrPAC study’s focus on molecular transducers, as well as the potential for MoTrPAC to revolutionize understanding of exercise’s physiological effects. While they value the study’s scientific advancements, their primary interest lies in the practical implications for patient care, hoping for specific exercise recommendations that could motivate patients. However, clinicians also expressed uncertainty about how the study’s findings will translate into their clinical settings, emphasizing the need for practical and easily applicable recommendations.

While some clinicians indicated curiosity about the molecular results of the study, most were more interested in the implications of the study for patient care and the practical outcomes, describing the need for real-world application and benefit to patients.

*“If it’s data that the outcomes don’t really impact how we take care of patients it’s just that it’s interesting to know from a molecular level*,* maybe it’s very interesting*,* but it’s not necessarily applicable. Whereas if there’s data that’s not just interesting from the physiologic standpoint*,* but also has an applicability to say*,* ‘Here’s how based on this data you should change your exercise prescriptions*,* how you approach exercise*,* with your patients exercise testing*,*’ and it’s very concrete and digestible data*,* then that’s the difference.” (P10*,* Male PCP)*.

These clinicians hoped that the study would provide some added specificity around exercise recommendations for patients. A few clinicians explained that having more specific recommendations and resources for patients may help motivate them to exercise.

*“It’s so hard to convince patients to do exercise. Every little bit helps. That’s how I would see it. What are the best conversations*,* what are the resources*,* what can we say*,* even if it increases the movements and exercises of only a handful of patients*,* that’s a victory for me.” (P05*,* Female PCP)*.

Some primary care practitioners were unsure how the study results would relate to their treatments/recommendations but were hopeful that there would be some impact.

*“I don’t know [how applicable it would be]. I would love to see it. […] I believe in this. I believe this is really important. I tried to incorporate this into my practice*,* and I’ll say I was frustrated because I didn’t feel like I had the tools or the specific information that was effective for most of my patients.[…] I never had molecular information on any of my patients*,* so I had no idea how the benefits of exercising were mediated at their tissue and cellular levels was just a guess. It’s a wild guess. If I had that information*,* and could provide it to patients*,* and help them get motivated to use and apply that information to follow up on it*,* would be marvelous. There’s a lot of steps in between there.” (P06*,* Female PCP)*.

One clinician valued the opportunity to better support patients with more information about exercise. Another was optimistic when considering the applicability of the MoTrPAC findings, noting they were “intrigued” by the notion of taking seemingly inaccessible information and turning it into practical applications.

Most emergency medicine physicians were unsure of the applicability of the study’s findings. They suggested adding considerations for acute care and demonstrating benefits to patients that may make findings more applicable to their practice.

*“Like so I’m not sure—I think in the emergency department*,* we do a lot of stress testing for cardiac*,* but I don’t think we do a ton of exercise recommendation and things like for patients. They would have to be very prescribed in terms of like*,* ‘These are the guidelines for physical activity*,*’ or*,* ‘these are the guidelines for what every human should be doing or what every 50-year-old should be doing.’ If it makes it to that mainstream*,* then I think that—and everybody knows*,* like*,* ‘Oh! These are the guidelines for this*,*’ and so if I’m seeing a patient who’s having complications of diabetes*,* or I’m seeing a patient with complications of obesity*,* I would know what the standard is and therefore potentially recommend whatever that standard is.” (P08*,* Female ED Clinician)*.

Clinicians emphasized the importance of brevity and directness in research dissemination. They then indicated how they would like the MoTrPAC findings to be packaged, and what they would like to learn from the study. Overall, clinicians emphasized the need for practical, accessible summaries of the findings disseminated through trusted medical channels while highlighting inquiries focused on ensuring the relevance and applicability of the study results to their clinical practice.

Clinicians were then asked how they would like to be informed about the MoTrPAC study. Most shared that a streamlined version of the findings focusing on practical applications would be the most impactful. A few clinicians emphasized the need for direct communication with providers through avenues such as listservs or partnering with organizations like the *American Academy of Family Physicians*,* American Medical Association*, or *American College of Cardiology* to enhance dissemination.

*“Typically*,* I find that if it’s vetted by an organization that I trust*,* like the American Academy of Family Practice*,* if they release it*,* like a link to it*,* in their news alert kind of thing*,* that’s typically how I like to get it. […] They typically will release when new guidelines are released or if a study that they think is going to be impactful for the patients*,* they put it on our radar with a release*,* yeah.” (P03*,* Male PCP)*.

One clinician described the process through which the *California Academy of Family Physicians* launches initiatives for family medicine practitioners, describing the impact of educational campaigns, virtual meetings, and creating opportunities for continuing medical education credits. Several explained that if the MoTrPAC results are published in the ‘right’ places (i.e., “relevant,” “well-established,” and “larger well-read” journals) and the information is relevant, the results will naturally “trickle down” to clinicians. Another shared that a combination of approaches would likely be more successful in spreading the information.

Clinicians in the study indicated what information would be helpful for their clinical practice and understanding of the data. These inquiries (Table 4) centered on usability, patient population, access, feasibility, provider education, clinical settings, outcomes, and specificity. From this, several strategies and tactics were developed for disseminating information (Table 5).

**Table 4:** MoTrPAC dissemination inquiries

Topic	Questions & Suggested Dissemination Content
Usability	• *“What is exercise testing at its very basic and what would one use it for?”*
	• *“[…] what would the barriers be or the activation threshold needed for a patient to be able to engage in that kind of testing?”*
	• *“For stress testing […] what are exclusions*,* what are inclusions*,* what are limitations?”*
Patient Population	• *“[…] how that translates to our specific patient population. […] One*,* would it make sense to our patients just at large*,* and two*,* does it make sense to our specific patients that we see here?”*
	• *I want to make a reminder that you like to be sure that in research*,* benefits those people who are most disadvantaged in our society. Not just the affluent and educated*,* but really those people who have the greatest health inequities*,* because of adverse social circumstances in their lives. How do you make this information available to them?”*
Access	• *“If insurance is to cover it*,* what is [the cost]*,* why?”*
	• *Are [patients] able to access these resources?”*
Feasibility	• *“How long does it take me to explain to someone what are the exercise that they need to do?”*
	• *How extensive is exercise testing*,* and what would be the overall impact?”*
Provider Education	• *“Am I really the expert to be telling people – is there a guideline where I can say*,* if you have back pain*,* these are the exercises that you should do.”*
	• *I would need to know specific recommendations for the results. Just getting them back would be important but knowing evidence-based recommendations when I get those results back*,* how to guide people*,* would be incredibly important.”*
Clinical Settings	• *“I think if there was some explanation about how different clinical settings could use the data that is placed forward*,* I think that would be cool to say that these are the molecular makers we found*,* and this is how it could be applicable to the emergency room. This is how it could be applicable to outpatient neurology*,* outpatient primary care*,* outpatient psychiatry.”*
	• *“Maybe like how exercise testing can be of a role or a value in post-hospitalized patients.”*
Outcomes	• *“What type of exercise testing is the most effective*,* and what are the results? What kind of results are we able to act upon*,* and how does that impact recommendations*,* and what is the strength of that recommendation*,* and how much benefit gets passed down to our patients if we are to engage in this?”*
	• *“This was a 12-week study. I think I would really like to know what they think the long-term benefits would be of the things that they found.”*
	• *“Something along the lines of maybe a dose-related phenomenon – like if you exercise this much or achieve this many metabolic equivalents for X duration*,* then this is the kind of benefit you can expect from it. You know*,* where it’d be easy to – in a way that it’d be relatively easy to explain to patients.”*
Specificity	• *“I’d like to know specific recommendations they have for each of the categories they were looking at like*,* “This is what we found was most helpful to change body composition positively. This is what we found was most helpful to increase endurance. This is what was most helpful to increase strength.” I would like solid evidence-based recommendations for the factors that I wanna target.”*
	• *“It would also be great if there would be a prescription that I would – that could be translated*,* like prescribing a medicine. You do five push-ups two times a day*,* something that I can actually write in the discharge instructions and that a patient can follow.”*
	• *“What are specific time recommendations*,* specific people*,* specific – people being patient population and specific like limitations?”*
	• *I think the big thing that I’d be looking for is how to provide recommendations on how to tailor the scientific recommendation and the everyday use*,* everyday layman’s terms for each individual patient.”*
	• *Well*,* I think if there were specific recommendations to give patients. Then*,* an easy way to maybe have it be part of their discharge instructions.”*

**Table 5:** Accelerating MoTrPAC discoveries to frontline clinicians: selected strategies and tactics

Theme	Strategy	Tactic
Equity and Inclusivity	Ensure that MoTrPAC knowledge contributes to mitigating health disparities and inequities	MoTrPAC discoveries presented at staff/faculty meetings at Federally Qualified Health Centers/Family Health Centers
Relevance to specific diseases	Clear elucidation for clinicians of how MoTrPAC discoveries in healthy individuals add insight into disease pathogenesis	Develop and fund future projects based on MoTrPAC (e.g., MoTrPAC Heart Failure, MoTrPAC Sickle Cell Disease, MoTrPAC Prehabilitation, MoTrPAC Arthritis, MoTrPAC Diabetes, etc.)
Updating clinical practice	Embed MoTrPAC lessons learned into clinic and hospital guidelines	Publish MoTrPAC CPET values in a timely manner in high-impact clinical journals and work to incorporate the new normal into clinical CPET laboratories.
Accessibility to CPET across a diverse population	Overcome payer inertia and practitioner unfamiliarity with analysis of how MoTrPAC discoveries can mitigate healthcare costs and disease burden.	Presentation of MoTrPAC clinical relevance and new normative data directly to third-party payers and large health systems (e.g., Kaiser, Banner Health, Intermountain, etc.)
Patient outreach	Utilize patient trust in primary care clinicians to disseminate MoTrPAC discoveries to the larger population	MoTrPAC develops pamphlets, blogs, short videos for clinician encounters in waiting rooms, etc.
Overcome clinician skepticism about the practical value of research on molecular mechanisms	Presentations of MoTrPAC discoveries must include examples of how research into molecular mechanisms led to novel therapies. Emphasize that MoTrPAC was designed to correlate phenotype with omics.	Relate therapeutic advances in which research on molecular mechanisms of disease to facilitate the development of novel therapies across a wide spectrum of human disease.
Improve novel research discovery access to frontline clinicians	Expand typical dissemination paradigms (academic journals, national research meetings) to websites, venues, podcasts, and listserv-based information opportunities.	Develop MoTrPAC-based pieces for online venues such as Med-IQ and UpToDate. Work with AMA and other organizations for online CMEs for relevant MoTrPAC publications. Create MOC opportunities based on MoTrPAC protocols.
Public health message	Optimize the role that physicians play in influencing policy through planned and thoughtful interactions with public agencies. MoTrPAC discoveries can serve as an effective discussion/presentation prompt.	Primary care physicians can emphasize the need to focus on physical activity to promote health in a variety of settings such as school boards, community health fairs, and interactions with public health agencies.

*Abbreviations* *MoTrPAC* Molecular Transducers of Physical Activity Consortium, *AMA* American Medical Association, *CME* continuing medical education, *MOC* maintenance of certification

## Discussion

This study conducted one-on-one semi-structured interviews among clinicians to (1) explore clinician experiences with and perceptions of exercise testing; (2) assess the research translation process as a whole; and (3) identify the perceived relevance of MoTrPAC discoveries and optimal ways to communicate these results to key stakeholders. We identified four overall themes from these semi-structured interviews: (1) exercise testing is an underutilized resource with many provider-level barriers to utilization; (2) patients face access and acceptability barriers to undergoing exercise testing; (3) despite low engagement with exercise research, many clinicians value exercise research and translating findings into clinical practice; and (4) clinicians value the potential applicability of MoTrPAC findings to their clinical practice, while emphasizing the need for clear and concise recommendations communicated directly to clinicians.

### Exposure to and understanding of exercise research

An important gap pointed out by clinicians was their limited understanding of the clinical utility of exercise testing and training, as well as limited exposure to exercise research. This gap highlights the ongoing dichotomy between advances in exercise-health research and clinical practice. For example, the American Heart Association has published a scientific statement [25] stating cardiorespiratory fitness – the predominant measure assessed during an exercise test – is potentially a stronger predictor of mortality than established risk factors such as smoking, hypertension, high cholesterol, and type 2 diabetes. Yet, cardiorespiratory fitness remains the only major risk factor not routinely assessed in clinical practice [25]. Therefore, simply acknowledging the benefits of exercise research, even by a major foundation such as the American Heart Association, without offering strategies for implementation into clinical care continues to expand translational research gaps.

This was emphasized by clinicians who identified major barriers to incorporating exercise testing in a clinical setting, including logistical considerations, provider knowledge, patient factors, and cost. Recommendations from the American Heart Association could mitigate some logistical considerations and provider knowledge; [26] which suggests most exercise tests can be supervised by appropriately trained non-physician health professionals (i.e., certified clinical exercise physiologists). However, although exercise tests are reimbursable by the Centers for Medicare and Medicaid Services and many private insurance companies, they must be supported with medical necessity using proper ICD codes [27]. Until ICD codes for exercise testing become a part of routine clinical care, there remains a major barrier to optimizing the use of exercise testing in a comprehensive approach for disease treatment and prevention.

However, the greatest potential barrier – even if exercise testing were ultimately incorporated into clinical care settings – is patient-level factors influencing whether or not someone becomes and remains physically active in order to achieve health benefits. Even if clinicians have the proper information to educate patients and prescribe exercise, if patients are not ready or motivated to make changes, the clinician’s recommendations will be disregarded. Thus, future research should not only aim to overcome feasibility and cost hurdles for implementing exercise testing in clinical settings, but also how to render exercise testing information to promote and motivate sustained behavior change among patients.

### Dissemination of MoTrPAC study findings into clinical practice

While clinicians valued the potential for MoTrPAC’s scientific advancements, their primary interest was in the practical implications for patient care. Unfortunately, much of scientific discovery is well-intended, yet the translation of scientific knowledge into clinical care remains to be desired. The greatest challenges to dissemination and implementation include limited resources, communication barriers, and resistance to change [28, 29]. Thus, disseminating and implementing findings from the MoTrPAC trial for clinical use will require a multi-faceted approach [28, 29]. 

**Fig. 1:**
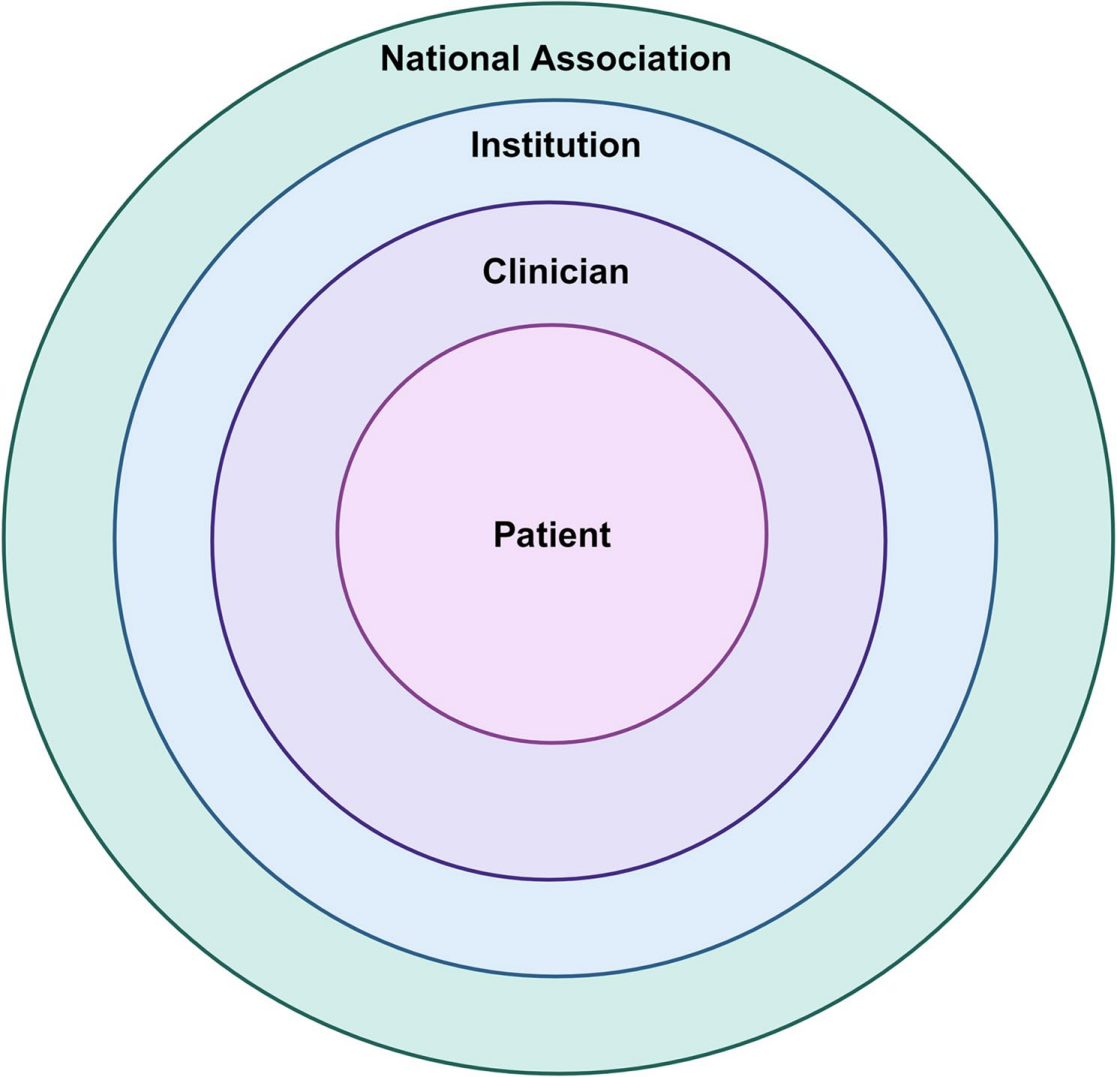
Socioecological dissemination framework. Created with BioRender.com

The first step in this approach will be to disseminate clear and accessible information to clinicians. The disseminated content should include (1) precision-level exercise recommendations physicians can prescribe to patients; and (2) easily digestible and interpreted material for patients to bring home from their clinician appointment. Some background information may be important to educate both clinician and patient on the importance of exercise and general PA guidelines. This content should be expanded upon for institution/organization-level conferences, symposia, medical journals, and media outlets. In addition, dissemination of precision-level findings dependent upon MoTrPAC results will need to be tailored based on a patients’ age, sex, race, and/or ethnicity. How these findings are disseminated will also be important [30–32]. MoTrPAC study findings should be summarized and disseminated with a socioecological structure encompassing a patient, with an identified population of interest or target audience within each level (Fig. 1) [33, 34]. Materials summarizing MoTrPAC findings should be created for the patient, the provider, chief executive officers or presidents of institutions, and the board of directors or president of national/international organizations [33, 35]. Examples of communication strategies, dependent upon the target audience, include coordinated symposia at national meetings, grand rounds and institutional-level symposia, media, town halls, and medical journals. Moreover, developing culturally relevant messaging is of importance to disseminate MoTrPAC findings through various forms of community engagement activities.

A second step of this approach will be to create opportunities for practice and feedback using implementation science [28, 29]. Once MoTrPAC findings are disseminated, fostering an environment of continuous learning and improvement through an implementation-based study will be of importance. This opportunity will allow clinicians to apply their knowledge of MoTrPAC findings and exercise into clinical practice, followed by feedback. Using an iterative and reflective approach, an implementation-based study could provide valuable feedback on how to best disseminate and implement MoTrPAC findings in a way that applies to clinical care and provides clinicians with more specific exercise recommendations that may help to motivate their patients to become physically active.

The final step of this multi-faceted approach will involve addressing provider-level and patient-level barriers [28, 29]. To optimally disseminate and implement MoTrPAC findings, there will need to be advocacy at the hospital and policy-levels to (1) expand the number of ICD codes that can be used to refer a patient for exercise testing; (2) have qualified exercise professionals available for consult with patients when providers have limited time and knowledge; and (3) expand access to exercise testing and assessments within a clinical setting. Moreover, advocacy and recommendations at the provider level, or from an exercise professional, could greatly impact patients and their intentions and motivations to adopt a physically active lifestyle [36, 37]. 

### Limitations

There are some limitations within this qualitative study. Convenience sampling was used, and thus, the sample is not randomized. Except for two, the clinician sample was primarily sourced from one site, which is already limited in sample size. Thus, experiences with research translation and exercise testing may be predominantly influenced by hospital and local policy, peers, and institutional culture. However, maximum variation sampling was used to gather heterogeneous perspectives regarding clinical role, gender, and race/ethnicity. Additionally, most clinicians in the sample did not have experience using exercise testing. Therefore, feedback regarding the understanding of and use of exercise testing may not apply to clinicians in other locations and settings across the country.

### Future directions

Given the exploratory nature of this study, more research is needed, primarily via large quantitative studies, to confirm these findings in the general population. Future work should determine how the implementation of exercise testing in primary care and other clinical settings could improve clinical outcomes, reduce healthcare expenditures, ensure the inclusiveness of diverse populations, and motivate patients to adopt and sustain exercise habits [38]. As outlined in Table 5, MoTrPAC investigators should develop dissemination strategies that utilize information venues currently popular with frontline clinicians such as Med-IQ and take advantage of continuing medical education (CME) and maintenance of certification (MOC) requirements by creating MoTrPAC-based content [39]. Additionally, MoTrPAC investigators should work with private and federal agencies to develop funding opportunities that extend the molecular and physiological mechanisms discovered in healthy individuals to address the diagnostic and therapeutic role of exercise in specific diseases and health conditions. Along with clinicians, additional key stakeholders such as patients, – adults and children most vulnerable to health impairments resulting from insufficient exercise – hospital and health network administrators, and third-party payor leaders should be included in a national learning health system in which MoTrPAC study findings are an integral data stream [40]. 

## Conclusions

There continues to be a gap between scientific discovery and translation and implementation into clinical practice. To better facilitate the rapid translation of discovery to benefit health, this exploratory qualitative analysis uncovered insights on exercise testing in clinical settings, identified beneficiary patient populations, and barriers to exercise testing. Highlighting the variable nature of research dissemination and implementation, these interviews stressed the need for a multi-faceted approach, including clear and concise summaries of MoTrPAC findings to be disseminated through trusted medical channels, providing opportunities for practice and feedback, and addressing provider-level and patient-level barriers. Findings from this qualitative research analysis are the first steps toward shaping the dissemination of valuable scientific discoveries from the MoTrPAC study.

## Supplementary Information

Supplementary Material 1.

Supplementary Material 2.

## Data Availability

The data (transcripts) that support the findings of this qualitative study are available from the Qualitative and Patient-Reported Outcomes Shared Resource (Q-PRO) of the Wake Forest Baptist Comprehensive Cancer Center, but restrictions apply to protect participant identity. Data are available from the corresponding author (katherine.collins-bennett@duke.edu) upon reasonable request and with permission of the Q-PRO.

## Abbreviations

AMA: American medical association
CAWG: Clinical applications working group
MoTrPAC: Molecular transducers of physical activity consortium
NIH: National institutes of health
PA: Physical activity
Q: PRO–Qualitative and Patient–Reported Outcomes
