# Supplementary material for: Accelerating the translation of findings from the MoTrPAC study to benefit clinical care: a qualitative analysis
Source: BMC Prim Care. 2025 Oct 27;26:324. doi: 10.1186/s12875-025-03030-6 (PMC12560478; doi:10.1186/s12875-025-03030-6)
Supplement: Supplementary file 1 — Supplementary Material 1. [file 12875_2025_3030_MOESM1_ESM.docx]

**MoTrPAC Interview Guide**

**Introduction**

- Thank you for your time today.
- Introduce self.
- The purpose of our conversation today is to help our study team learn from your experience as a clinician. We want to hear your perspective about how research study findings may impact your clinical practice and how research findings should be packaged to be best understood and applied by providers like you.
- As a clinician in this area, we consider you to be the expert. Anything you choose to share, both positive and negative, is highly valuable.
- This interview will last no longer than thirty minutes and I will audio record our conversation so we can go back and be sure to document your feedback. If at any point, you would like to take a break, skip a question, ask a question, or stop the interview, just let me know. Can I confirm I have your permission to record the interview?
- Do you have any questions for me before we start?

Today we’ll be talking about the Molecular Transducers of Physical Activity Consortium (MoTrPAC). To provide a bit of background on MoTrPAC, the consortium aims to understand how exercise improves and maintains health at the molecular level. To do so, healthy adults were recruited and engaged in either a 12-week aerobic training program or a 12-week strength training program. For today, we’re interested in a particular outcome of the study, which are physical fitness effects as measures by cardiopulmonary exercise testing (CPET). One of the major goals of the consortium is to enable clinicians to make more specific exercise recommendations to patients when using exercise as an intervention to improve health. This is why we would like to talk to you today.

To begin,

1. What are your initial thoughts about the MoTrPAC study?

**Clinical Practice**

Next, I want to understand how you use exercise testing in your clinical care.

1. How often do you often refer patients for exercise testing?
2. How useful is exercise testing for your treatment of patients?
3. What barriers do you see to including exercise testing in patient care?
   1. Reimbursement/fees/cost structure, access

**Research Engagement**

Now I’m going to ask you a few questions about your thoughts on exercise testing research.

1. What are your feelings on exercise testing research?
   1. Accessible, understandable, inclusive
2. As a clinician, how important is it to you to familiarize yourself with current exercise testing research?
3. How do you typically find out about new research?
   1. Journals, professional societies, conferences, Google, social media, grand rounds, etc.
4. How would you like to find out about the results of the MoTrPAC study?

**Incorporating Research into Clinical Practice**

1. What can researchers do to make exercise research more easily incorporated into clinical practice?
   1. What are the facilitators to incorporating research findings into your clinical practice?
2. What are the barriers to incorporating research findings into your clinical practice?
3. How well do you think the MoTrPAC study will produce research findings that will be applicable to your clinical care?

What else would you like to add before we wrap up that you feel may help us to better understand clinicians’ perceptions of MoTrPAC or the application of exercise research within clinical practice?

Thank you for sharing your thoughts and experiences today.
