# Supplementary material for: Accelerating the translation of findings from the MoTrPAC study to benefit clinical care: a qualitative analysis
Source: BMC Prim Care. 2025 Oct 27;26:324. doi: 10.1186/s12875-025-03030-6 (PMC12560478; doi:10.1186/s12875-025-03030-6)
Supplement: Supplementary file 2 — Supplementary Material 2. [file 12875_2025_3030_MOESM2_ESM.pdf]

WAKE FOREST
UNIVERSITY
HEALTH SCIENCES

Office of Research
INSTITUTIONAL REVIEW BOARD

MEMORANDUM

To: Erin Sutfin
Public Health Sciences-Social Sciences

From: Brian Moore, Chair
Institutional Review Board

Date: 8/31/2023

Subject: Not Human Subjects Research: IRB00100858
Accelerating the Translation of MoTrPAC Data to Benefit Clinical Care - An Exploratory Study, Qualitative Interaction with Frontline Clinicians

The Wake Forest University School of Medicine Institutional Review Board has reviewed your protocol and determined that it does not meet the federal definition of research involving human subject research as outlined in the federal regulations 45 CFR 46. 45 CFR 46.102(f) defines human subjects as “a living individual about whom an investigator (whether professional or student) conducting research obtains (1) data through intervention or interaction with the individual, or (2) identifiable private information.”

The information you are receiving is not individually identifiable. In recent guidance published by the Office of Human Research Protections (OHRP) on the Guidance on Research Involving Coded Private Information or Biological Specimens, OHRP emphasizes the importance on what is being obtained by the investigator and states “if investigators are not obtaining either data through intervention or interaction with living individuals, or identifiable private information, then the research activity does not involve human subjects.”

This submission has met the requirements of the 2019 Common Rule.

Note that only the Wake Forest University School of Medicine IRB can make the determination for its investigators that a research study does not meet the federal definition of human subject research. Investigators do not have the authority to make an independent determination that a study does not meet the federal requirements for human subject research. Each project requires a separate review and determination by the Board. The Board must be informed of any changes to this project, so that the Board can determine whether it continues to not meet the federal requirements for human subject research. If you have any questions or concerns about this information, please feel free to contact our office at 716-4542.

The Wake Forest School of Medicine IRB is duly constituted, has written procedures for initial and continuing review of clinical trials; prepares written minutes of convened meetings, and retains records pertaining to the review and approval process; all in compliance with requirements of FDA regulations 21 CFR Parts 50 and 56, HHS regulations 45 CFR 46, and International

Conference on Harmonisation (ICH) E6, Good Clinical Practice (GCP), as applicable. WFSM IRB is registered with OHRP/FDA; our IRB registration numbers are IRB00000212, IRB00002432, IRB00002433, IRB00002434, IRB00008492, IRB00008493, IRB00008494, and IRB00008495.

WFSM IRB has been continually fully accredited by the Association for the Accreditation of Human Research Protection Programs (AAHRPP) since 2011.
